# The misuse of insulin by males with Type 1 Diabetes Mellitus for weight and/or shape control: a systematic scoping review

**DOI:** 10.1007/s40200-022-01151-8

**Published:** 2022-11-15

**Authors:** Georgia Goddard, Melissa Oxlad, Deborah Turnbull

**Affiliations:** 1 School of Psychology, The University of Adelaide, Adelaide, Australia; 2 Freemason’s Centre for Men’s Health and Wellbeing, Adelaide, Australia

**Keywords:** Males, Type 1 diabetes mellitus, Insulin restriction and/or omission, Disordered eating behaviours, Scoping review

## Abstract

**Background:**

Insulin restriction and/or omission in Type 1 Diabetes Mellitus (T1DM) is a risky disordered eating behaviour increasingly reported in the literature. Most existing literature has focused on females with T1DM. Previous research, however, suggests disordered eating behaviours are over-represented in males with T1DM versus males without T1DM. This systematic scoping review sought to summarise the existing literature to contribute to the development of an understanding of males’ misuse of insulin for weight and/or shape control.

**Methods:**

A systematic scoping review methodology was used. We searched six electronic databases. Eligible articles were quantitative, qualitative, or mixed methods empirical studies with primary data on the restriction and/or omission of insulin for weight and/or shape control among males, written in English from database inception to December 2021.

**Results:**

A final sample of 56 articles was included (48 quantitative, six qualitative and two mixed methods). Where it was reported to occur, estimates of the behaviour ranged from 5 to 75% in females and 1.4% to 76% in males. In 12 studies examining sex differences, only three reported higher prevalence in females. Understandings about the behavioural context of the behaviour were hampered by lack of qualitative research (n = 4 studies).

**Conclusion:**

Despite common preconceptions, this disordered eating behaviour appears to occur similarly in males and females. The findings shed light on areas for future research, including aetiology in males and the need for longitudinal and qualitative research. Clinical recommendations include being alert to the possibility of this behaviour in males with Type 1 Diabetes Mellitus and the need for health professionals to use open-ended questions to explore current and past deliberate restriction and/or omission of insulin among their patients.

**Supplementary Information:**

The online version contains supplementary material available at 10.1007/s40200-022-01151-8.

## Introduction

Disordered eating and eating disorders appear alarmingly high in individuals with Type 1 Diabetes Mellitus (T1DM) [1–3]. This is concerning given that disordered eating behaviours in individuals with T1DM increase the risk of morbidity and mortality [3–5]. A unique, non-dietary, purging behaviour available only to individuals with T1DM or Type 2 Diabetes Mellitus (T2DM) dependent on insulin therapy is the deliberate restriction and/or omission of insulin, inducing hyperglycaemia and weight loss [6–9].

While a clear diagnostic term and diagnostic criteria are currently lacking [10], individuals deliberately restricting and/or omitting insulin for weight and/or shape control have adopted the term "diabulimia" to describe their behaviour and report a distinct 'diabulimic' identity separate from other eating disorder labels [11]. While it is important to explore and respect terminology of those with lived experience, controversy around the term ‘diabulimia’ exists as it is often used differently between studies creating confusion regarding its meaning. Some researchers use the term to describe insulin restriction and/or omission for weight and/or shape control, while others use it to describe eating disorders comorbid with T1DM more broadly [12]. It has also been argued that the term is too narrow, predominantly represents bulimic symptomology and does not capture the broad spectrum of disturbed eating behaviour seen in diabetes [12–14]. Therefore, in the absence of a diagnostic term, we refer to the deliberate restriction and/or omission of insulin to influence weight and/or shape.

While insulin restriction and/or omission can occur for various reasons beyond weight and/or shape control (e.g., forgetfulness, avoidance of painful injections etc.), insulin restriction for weight and/or shape control" is suggested to occur in 4 to 58% of people with T1DM [14]. Highlighting the seriousness of deliberately restricting and/or omitting insulin, research has found that when compared to other weight control methods, it led to the highest rates of retinopathy and nephropathy [4, 15], increased the risk of mortality by 3.2 times and reduced average life span by 14 years [4].

Despite its growing interest to researchers and its clinical significance, deliberate insulin restriction and/or omission for weight and/or shape control remains an understudied problem with females being consistently over-represented in existing research [14]. As a result, much less is known about males’ disordered eating behaviours. A lower incidence of eating disorders reported in males generally may contribute to the paucity of research investigating disordered eating behaviours in males with T1DM [16]. Although recent prevalence rates for eating disorders in males are likely a gross underestimate [17], research has demonstrated that males are at risk of eating disorders and with comparable levels of distress and disability to females [18, 19]. Furthermore, the prevalence of eating disorders among males has increased dramatically over the last two decades [20], and emerging evidence suggests that disordered eating behaviour in males may be increasing faster than in females [19].

Importantly, research has demonstrated that compared to males without diabetes, disordered eating behaviours are still overrepresented in males with T1DM [7, 21]. Research has also reported high levels of body dissatisfaction and drive for thinness among males with T1DM [21–23]. Given differences in prescribed sociocultural body ideals for males and females [24], which have implications for the associated maladaptive behaviours individuals may undertake to achieve such ideals [25, 26], it is reasonable to assume differences in disordered eating behaviours may exist among males and females with T1DM.

A preliminary search of MEDLINE, the Cochrane Database of Systematic Reviews and Johanna Briggs Institute Evidence Synthesis confirmed that no published systematic or scoping reviews concerning the deliberate misuse of insulin among males with T1DM for weight and/or shape control existed. A manual search of grey literature yielded one systematic review exploring insulin restriction and/or omission for weight and/or shape control in males across *n* = 16 studies; this was part of an unpublished dissertation [27].

We aimed to identify and summarise existing knowledge about the misuse of insulin among males with T1DM for weight and/or shape control to answer the following primary review questions:

1. What is the nature of males’ misuse of insulin for weight and/or shape control, including population characteristics?
2. What influences males’ misuse of insulin for weight and/or shape control?
3. What are the impacts of males’ misuse of insulin for weight and/or shape control?
4. What gaps exist in the current literature about insulin misuse for weight and/or shape control among males?

## Method

### Design and search strategy

A systematic scoping review methodology was employed using the the Joanna Briggs Institute [28] guidelines and the Preferred Reporting Items in Systematic Reviews and Meta-analyses guidelines (PRISMA-ScR; [29]; see Supplementary Table 1). The priori protocol was registered on Open Science Framework (removed for blind review). The search strategy aimed to locate published and unpublished studies. Six databases (Psychinfo, PubMed, Embase, Web of Science, CINAHL and Scopus) were searched for relevant studies from database inception to December 2021. Initially, preliminary searches were undertaken across the databases to identify relevant subject headings and keywords. The final search strategies were finessed in collaboration with an experienced research librarian (See Supplementary Table 2) and database searches were supplemented with a manual search of reference lists of included studies.

### Eligibility criteria and study selection

We used the Population, Concept, and Context (PCC) framework [28] to conceptualise the research questions as follows.

- Participants: Studies that include male participants, of all ages, with T1DM deliberately restricting and/or omitting insulin.
- Concept: Deliberate restriction and/or omission of insulin for weight and/or shape control.
- Context: Any geographic setting.

Inclusion criteria were quantitative, qualitative, mixed methods and case studies published from database inception to December 2021. Studies were included if they: (i) investigated insulin misuse for weight and/or shape control or within the context of disordered eating, (ii) investigated males with T1DM (studies investigating males and females were eligible for inclusion if data about males was reported separately; studies investigating males with T1DM and Type 2 Diabetes Mellitus (T2DM) were eligible for inclusion if data about males with T1DM was reported separately), (iii) presented primary data, and (iv) were written in English. This scoping review considered experimental and quasi-experimental study designs, analytical observational studies (prospective and retrospective cohort studies, case–control studies and analytical cross-sectional studies) and descriptive observational designs (descriptive cross-sectional studies, individual case reports and case series) for inclusion. Qualitative studies using designs such as phenomenology, grounded theory, ethnography, thematic analysis, and qualitative description were also eligible. Relevant unpublished studies or grey literature such as reports, dissertations and theses were also considered.

Excluded studies were those not written in English, abstracts, editorials or opinion pieces, discussion or review articles not reporting primary data, and studies using a comparator (e.g., females) that did not present male data separately. Studies were also excluded if they investigated insulin restriction and/or omission for reasons other than for weight and/or shape control (e.g., forgetfulness or avoidance of pain).

**Fig. 1:**
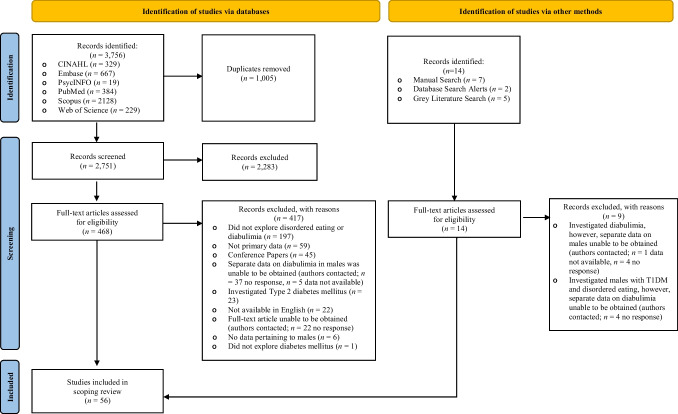
PRISMA flow diagram [29] demonstrating the article selection and screening process

The database searches identified 3,756 potentially eligible studies. A further 14 studies were sourced manually from the reference lists of database-identified articles, resulting in 3,770 studies for consideration (see Fig. 1). All identified references and abstracts were pooled into EndNote X9 software to remove duplicates, screen for titles and abstracts and full-text in detail based on the predetermined eligibility criteria. The first and second authors co-screened a random subset of 10% of potentially eligible studies (interrater agreement 99%, *K* = 0.97, *p* < 0.05), with any discrepancies resolved by consensus discussion.

The findings of the included articles were extracted by the first author and cross-checked by the second author. Extracted data included research setting/country, year of publication, study design, sample size and characteristics, key findings on males’ experiences of insulin restriction and/or omission and any relevant limitations or biases.

## Results

### Description of included studies

The final sample comprised 53 peer-reviewed studies (published between 1982 and 2021) and three unpublished dissertations, representing 46 samples; 48 studies reported quantitative data, six studies reported qualitative data, and two studies were mixed methods (Table 1). Most studies originated from high-income countries (United States of America (*n* = 10), the United Kingdom (*n* = 9) or Norway (*n* = 9)).

**Table 1:** Characteristics of Included Studies (N_studies_ = 56)

Lead Author (Year)	Country	Study Aim	T1DM Sample Size (*N*_*studies*_ = *45)*		Study Methodology (Study Design & Data Collection)	Recruitment Source	Key Outcomes
			Total (*N)*	Males (*N* or %)			
Quantitative studies:							
Ackard* (2008)	United States	To compare the prevalence of DEB and body dissatisfaction between adolescents with T1DM and a population- based sample of youth	143	73	Cross-sectional Self-report survey with custom question about insulin restriction/omission in the past year, and medical charts	Look AHEAD (Action for Health in Diabetes) Study Clinic-based sample	Of those who reported using to “lose weight” or “keep from gaining weight”; -1.4% (*SE* = 1.4) restricted insulin -1.4% (*SE* = 1.4) omitted insulin
Altinok (2017)	Turkey	To show the reliability and validity of a Turkish version of the DEPS-R in children and adolescents with T1DM	200	90	Cross-sectional Self-report survey with the DEPS-R (Turkish) and physical measurements	Routine Medical Visit Clinic-based sample	Of those who had a DEPS-R score of 20 or higher in the past 28 days; -37.5% restricted insulin -25% omitted insulin
Apergi (2020)	Greece	To translate and adapt the DEPS-R questionnaire into the Greek language	100	30	Cross-sectional Self-report questionnaires with the DEPS-R (Greek) and medical records	Outpatient Diabetes Clinics of the "Attikon" and "Laiko" general hospitals Clinic-based sample	Over the previous 28-days -13 males (43.4%) restricted insulin (*M* = 1, *SD* = 1.62, 0 Never – 5 Always) -7 males omitted insulin as well (*M* = 0.53, *SD* = 1.31, 0 Never – 5 Always)
Araia* (2017)	Australia	To examine gender differences in DEB and body dissatisfaction in adolescents with T1DM	477	180	Cross-sectional MY-Q (adapted) from a national online survey	The Diabetes MILES (Management and Impact for Long-term Empowerment and Success) Youth-Australia Study Population-based sample	Over the past 14-days, -26 males misused insulin 1–3 days (approximately 14.4%) -7 males misused insulin 4 or more days (approximately 3.8%)
Araia* (2020)	Australia	To explore potential risk and protective psychological correlates for DEB in youth with T1DM by considering a multitude of general and diabetes-specific risk factors, including diabetes distress and diabetes- related resilience			Cross-sectional DEPS-R from a national online survey		Over the past 28 days, -127 males (70.56%) engaged in insulin restriction (*M* = 1.32, *SD* = 1.236, 0 Never – 5 Always) °53 (Never) – 29.44% °60 (Rarely) – 33.33% °40 (Sometimes) – 22.22% °13 (Often) – 7.22% °11 (Usually) – 6.11% °3 (Always) – 1.67% -53 (29.4%) males engaged in insulin restriction (M = 0.19, SD = 0.508, 0 Never – 4 Often) °153 (Never) – 85% °20 (Rarely) – 11.11% °6 (Sometimes) – 3.33% °1 (Often) – 0.56% °0 (Usually) °0 (Always)
Bächle [sic] (2016)	Germany	To estimate the prevalence of DEB and insulin restriction in youths with early-onset T1DM of long duration and assess associations between disordered eating/insulin restriction and metabolic outcomes	819	414	Cross-sectional Postal self-report questionnaire with a custom question on the frequency of insulin restriction and the number of carbohydrate exchange units consumed without insulin coverage	Diabetes Care Teams and the German Diabetes Centre Population-based sample	Over the past 7 days -22 (5.3%) males were SCOFF negative and deliberately restricted their insulin °*M* carbohydrate units without insulin coverage per week was 13.2 (SD = 10.6) -8 (1.9%) males were SCOFF positive and deliberately restricted their insulin °*M* carbohydrate units without insulin coverage per week was 18.8 (SD = 18.6)
Baechle (2014)	Germany	To compare the prevalence of symptoms of DEB in intensely treated male and female patients with early-onset and long-duration diabetes with representative peers in Germany using large population-based samples and to assess insulin restriction in T1DM	629	54.1%	Case–Control Standardised, Self-Report Questionnaire Custom question on the frequency of insulin restriction and omission after carbohydrate intake with predefined reply options	German Nationwide, Population-based Cohort Study Population-based sample	Across the past 7 days -18.5% of males restricted insulin at least 3 times per week -6% of males restricted insulin more than five times per week SCOFF-positive males reported insulin restriction (> 5 times / > 3 times) significantly more frequently than SCOFF-negative patients (15.8%/36.8% vs. 4.8%/16.4% respectively, *p* = .018/.003)
Baechle (2019)	Germany	To analyse the prevalence and course of DEB in adolescents with intensively treated T1DM, describe differences in age-specific DEB symptoms, and identify predictors of current DEB	1,318	584	Longitudinal Cohort Standardised, self-report SCOFF questionnaire (modified)—fifth question replaced with a question on frequency of insulin restriction and omission after carbohydrate intake	German Nationwide, Population-based Cohort Study Population-based sample	Across the past 7 days, -47 (8.2%) males omitted insulin °14 (48.3%) with disordered eating behaviours °33 (6%) without disordered eating behaviours
Beam (2021)	United States	To examine whether and how emotion dysregulation and depressive symptoms are associated with insulin omission among late adolescents with T1DM	236	39% (*n* = 91)	Cross-sectional Online self-report survey with custom questions about the frequency of insulin restriction/omission and the frequency of insulin restriction/omission for the specific purpose of losing weight with Likert-scale answers and physical measurements,	Paediatric Endocrinology Clinics in two Southwestern US cities Clinic-based sample	*M* frequency of insulin restriction and/or omission for losing weight among males was 0.1630 (SD = .37143; 0 Never – 5 Always
Bryden* (1999)	United Kingdom	To examine disordered eating, insulin misuse, weight change, and their relationships with glycemic control and diabetic complications in adolescents with T1DM followed up over eight years	76	39	Longitudinal Cohort – Follow Up Standardised investigator-based EDE (adapted) and physical measurements	Follow-up assessment of a cohort initially recruited from a case register of a hospital outpatient clinic Clinic-based sample	No males reported restricting or omitting insulin past or present at follow-up
Cherubini (2018)	Italy	To evaluate the association of clinical, metabolic, and socioeconomic factors with DEB among adolescents with T1DM	163	48.5%	Cross-sectional DEPS-R Self-Report Questionnaire and Clinical Database	The Italian Insulin-Dependent Diabetes Registry (RIDI) Population-based sample	Over the previous 28 days -4 males indicated insulin misuse, of whom 1 had a score of 20 > on the DEPS-R
Colton (2015)	Canada	To characterize the clinical presentation of individuals with T1DM seeking treatment at an academic eating disorder treatment centre, and to assess the relative effectiveness of an intensive, CBT-based day hospital treatment program for individuals with T1DM	100	2	Retrospective Chart Review Clinical Charts 1990–2012 and Information from EDE diagnostic interview	Ambulatory Care for Eating Disorders Program at the Toronto General Hospital Clinic-based sample	No males reported restricting or omitting insulin
D'Emden* (2012)	Australia	To compare the YEDE-Q and EDI-3RC against the chEDE	124	58	Cross-sectional YEDE-Q (adapted) and EDI-3 Risk Composite (adapted) self-report questionnaires, the ChEDE diagnostic interview	Queensland Diabetes Centre Clinic-based sample	Over the previous 28 days -3.4% of males reported insulin misuse (n = 2)
D'Emden* (2013)	Australia	To describe the presence and types of disturbed eating behaviours and thoughts in a combined male/ female Australian sample of adolescents with T1DM					
Fairburn (1991)	United Kingdom	To determine the prevalence of clinical eating disorders and lesser degrees of DEB in young adults with IDDM and a matched sample of non-diabetic female controls	100	46	Cross-sectional EDE semi-structured Interview	Case Register of an Oxford Clinic Clinic-based sample	Over the previous 28 days, no males reported restricting or omitting insulin
Grylli (2004)	Austria	To investigate the prevalence and clinical manifestations of clinical eating disorders and sub-threshold problems of eating and shape among Austrian adolescents with T1DM	251	103	Cross-sectional Self-Report Screen and EDE Semi-Structured Diagnostic Interview for those who scored above the screening cut-off	List of Current Patients from 10 Outpatient Clinics for Diabetes Care Clinic-based sample	Over the previous 28 days, no males reported restricting or omitting insulin
Herpertz* (1998; 55)	Germany	To explore the prevalence of clinical and subclinical eating disorders, the extent of intentional omission of insulin and oral antidiabetic agents, and its relationship to glycaemic control in an inpatient and outpatient population of males and females with T1DM and T2DM	341	153	Cross-sectional FSE (adapted) self-report questionnaire, medical records and SIAB (adapted) standardised interview	12 Diabetes Centres in two German cities Clinic-based sample	1 male was found to engage in intentional insulin omission (according to the FSE)
Herpertz* (1998; 56)	Germany	To assess prevalence of eating disorders with IDDM and NIDDM					
Justice (2004)	United States	To compare T1DM and T2DM comorbidity for eating disorders in adolescents	143	72	Cross-sectional EDI (adapted) self-report questionnaire, EDE structured interview, 24-h recall interviews and medical charts	The Paediatric Endocrinology Clinic at the University of Florida Clinic-based sample	No males reported insulin manipulation
Lawrence (2008)	United States	To describe the weight-loss practices and weight-related issues reported by youth with diabetes, according to sex and diabetes type	2837	1422	Cross-sectional Self-report questionnaire with a custom question about methods to lose weight including skipping insulin doses, physical measurements, and blood samples	SEARCH for Diabetes in Youth Study Population-based sample	6 males (1.3%) who indicated they had tried to lose weight had omitted insulin to do so
Mannucci (1995)	Australia/China	To describe eating attitude and behaviour in IDDM patients	118	56	Case–Control BITE and EAT-26 self-report questionnaires and structured interview	Not reported	No males reported insulin manipulation
Markowitz (2010)	United States	To update and validate the DEPS in contemporary youth with T1DM	112	49	Cross-sectional DEPS-28 and clinician-rated self-report questionnaires and medical records	Tertiary Care Centre Clinic-based sample	Over the previous 28 days -24% of males reported insulin manipulation
Matthews (2019)	United Kingdom	To investigate prevalence rates of insulin misuse by adults with T1DM, and to explore the relationships between insulin misuse and gender, eating disorder psychopathology and diabetes-related distress	219	47	Cross-sectional Custom self-report insulin questionnaire	Advertisements Published on Online Diabetes Support Communities, Social Media Platforms Population-based sample	Over the previous 7 days -1 (2.1%) males reported insulin restriction for weight control -2 (4.3%) male reported insulin restriction for weight loss -1 (2.1%) male reported insulin omission for weight control -1 (4.3%) reported insulin omission for weight loss
Meltzer (2001)	United States	To examine the relationship between disordered eating attitudes and behaviours, BMI, and glycaemic control in adolescents with T1DM	152	54	Cross-sectional EDI (adapted) self-report questionnaire and physical measurements	Diabetes Specialist Clinics at the University of Florida Health Science Centre and Nemours Children's Clinic Clinic-based sample	4 males (13.5%) reported skipping or manipulating insulin doses
Neumark-Sztainer* (2002)	United States	To examine the prevalence of specific weight control practices/ DEB and associations with sociodemographic characteristics, BMI and weight perceptions, family functioning, and metabolic control among adolescent females and males with T1DM	143	73	Cross-sectional Self-report survey with the DEPS and custom question on skipping insulin doses or taking less insulin than prescribed	Diabetes Clinic at Children's Hospital in St Paul; AHEAD Study Clinic-based sample	Of those who reported using to “lose weight” or “keep from gaining weight” in past year -1 (1.4%) male reported restricting insulin -1 (1.4%) male reported omitting insulin
Peducci (2018)	Italy	To investigate DEB and eating patterns in the context of a teenage population with T1DM	85	43	Cross-sectional EDE (adapted) clinician-administered semi-structured interview and physical measurements	Regional Diabetes Center of the University Children Hospital Clinic-based sample	Over the past 28 days -2 males (5.8%) reported insulin misuse
Peveler* (1992)	United Kingdom	To determine the prevalence of clinical eating disorders and lesser degrees of DEB in adolescents with IDDM and a matched sample of nondiabetic control subjects	76	43	Cross-sectional EDE (adapted) clinician-administered semi-structured interview, physical measurements and	Case register of a hospital outpatient clinic Clinic-based sample	No males reported current or past insulin omission or insulin restriction
Phillipi (2013)	Brazil	To evaluate the frequency of risk behaviours for eating disorders in patients with T1DM and their association with gender, nutritional status, variables related to T1DM, and body satisfaction	189	48	Cross-sectional EAT (Portuguese) and BITE self-report questionnaires and physical measurements	3 Institutions Specialising in Diabetes Care in the City of Sao Paulo Clinic-based sample	No males reported insulin manipulation
Pinhas Hamiel (2013)	Israel	To use clinical and laboratory criteria to create an algorithm to assist in the detection of intentional insulin omission	287	155	Cross-sectional HbA1c levels DSM-IV	Juvenile Diabetes Center in Maccabi Health Care services Clinic-based sample	No males reported insulin manipulation
Powers (1990)	United States	To assess prevalence of eating disorder among sample of adolescent diabetes patients	97	51	Cross-sectional Eating Habits Self-Report Questionnaire (adapted)	The University of South Florida Diabetes Center Clinic-based sample	Over previous 7 days -4.1% of males withheld insulin
Pursey (2020)	Australia	The aim of this study was to evaluate a range of DEB in adolescents reporting a diagnosis of diabetes compared to their non-diabetic peers in a large community-based population sample of Australian adolescents, as well as to evaluate DEB according to insulin restriction in adolescents reporting diabetes	52	31	Secondary Data Analysis of a Longitudinal Investigation Online self-report questionnaire with a custom question assessing the frequency of insulin restriction as a way of controlling shape and weight	EveryBODY Study Population-based sample	Based on 31 males, the mean frequency of insulin restriction over the previous 28 days was 2.90 (*SD* = 7.245; min = 0 and max = 28)
Rancourt (2019)	United States	To examine associations among constructs included in the recently proposed T1DM specific modified dual pathway model and examined age as a moderator of these associations	818	342	Cross-sectional Medical Charts, DEPS-R self-report questionnaire	Email invitation from the T1DM Exchange Clinic Network Clinic-based sample	Over the previous 28 days, -49 males reported omitting insulin (approximately 14.32% °28 teenage males °17 young adult males °4 adults
Rose (2020)	United States	To evaluate a model of risk for engaging in DEB in adolescents with T1DM with the goal of identifying youth at risk for DEB and mechanisms by which negative affect might be associated with DEB in adolescents with T1DM	100	55	Cross-sectional Online self-report DEPS-R, parent-report questionnaire	Screening of Clinic Schedules of Diabetes Clinics in a Metropolitan Paediatric Hospital Clinic-based sample	Over the previous 28 days °-53 males responded to Item 4 (“When I over- eat, I do not take enough insulin to cover the food”) °*M* score of 1.13 (*SD* = 1.093; Min 0, Max: 4) °-52 males responded to Item 13 (“After I overeat, I skip my next insulin dose.”) °*M* score of 0.27 (*SD* = *.*819; Min: 0, Max:4)
Saßmann (2015)	Germany	To examine the psychometric properties of the DEPS-R in a sample of young people with T1DM	246	125	Cross-sectional DEPS-R (German) self-report questionnaire and classification of expect panel	6 Paediatric Diabetes Centres in Germany Clinic-based sample	3 (2.4%) males were suspected to practice insulin restriction by an expert panel
Svennson (2003)	Sweden	To investigate eating behaviour in adolescent males with IDDM and compared it with that in age-matched healthy male control subjects	109	109	Case-controlled EDI-C (adapted) self-report questionnaire, physical measurements, and clinician-administered interview	Clinics across Central Sweden Clinic-based sample	3 males reported insulin restriction on the self-report questionnaire (approximately 2.75%) -1 with a high score of the EDI-C drive for thinness subscale All males denied insulin restriction in the interview
Troncone (2020; 63)	Italy	To examine body image problems and their associations with disordered eating behaviour in adolescents with T1DM and well-matched healthy peers	183	85	Cross-sectional Brief interview, physical measurements, the DEPS-R self-report questionnaire and a parent-report measure	Patients from a Southern Italian Centre for Children and Adolescents with T1DM Clinic-based sample	Over the past 28 days -53 males (28.9%) reported insulin restriction -17 males (9.3%) reported insulin omission
Troncone (2020; 64)	Italy	To examine associations of DEB with body image problems, parents’ eating disorder symptoms, and emotional and behavioural problems among adolescents with T1DM	200	102	Cross-sectional Brief interview, physical measurements, the DEPS-R self-report questionnaire and a parent-report measure	Patients from a Southern Italian Centre for Children and Adolescents with T1DM Clinic-based sample	Over the past 28 days -23 males (11.5%) reported insulin omission -63 (31.5%) males reported insulin reduction
Urakami (2000)	Japan	To examine the prevalence and clinical features of patients with eating disorders among T1DM	100	44	Cross-sectional Structured Interview and Self-Report Questionnaire	Not reported	No males reported insulin manipulation
Wilson (2015)	United Kingdom	To examine risk factors for eating disturbance in young people with T1DM	50	20	Cross-sectional ChEDE semi-structured interview and medical files	2 Paediatric Clinics in the East of England Clinic-based sample	No males reported insulin manipulation across the past 28 days
Wisting* (2016)	Norway	To investigate associations between HbA1c and eating disorder psychopathology, coping strategies, illness perceptions, and insulin beliefs in young males and females with T1DM and to assess the extent to which these variables explain the variance in HbA1c	105	44	Cross-sectional ChEDE (adapted) structured interview	Norweigan Childhood Diabetes Registry (NCDR) Population-based sample	No cases of insulin restriction due to weight- or shape concern among males across the past 28 days
Wisting* (2015)	Norway	To investigate correlates of eating disorder psychopathology in adolescent males and females with T1DM					
Wisting* (2017)	Norway	To investigate eating patterns among male and female adolescents T1DM and the associations with age, zBMI, eating disorder pathology, intentional insulin omission, and metabolic control					
Wisting* (2013; 50)	Norway	To establish the prevalence of DEB and insulin omission among adolescents with T1DM using intensive insulin treatment in a nationwide population-based study	770	380	Cross-sectional DEPS-R (Norwegian) self-report questionnaire and clinical data from the NCDR	NCDR Population-based sample	Over the past 28 days °-9.4% of males restricted insulin after overeating °-4.5% of males skipped insulin because of overeating
Wisting* (2013; 74)	Norway	The purpose of this study was to examine the psychometric properties of the DEPS-R (Norwegian version) in a large sample of young patients with T1DM, to establish norms, and to validate it against the EAT-12					
Wisting* (2019; 53)	Norway	To investigate the impact of eating disorder psychopathology, illness perceptions, insulin beliefs, and coping strategies on metabolic control in adults with T1DM, with a specific focus on gender differences	282	112	Cross-sectional DEPS-R (Norwegian) self-report questionnaire and clinical data from the NCDR	NCDR Population-based sample	Over the past 28 days -85 males (76%) endorsed insulin restriction (Q4) a 1 or more °*M* score of 1.43 (*SD* = 1.198, 0–5) -5 males (4%) endorsed insulin omission (Q13) a 1 or more °*M* score of 0.05 (*SD* = .208, 0–5)
Wisting* (2020)	Norway	To investigate the impact of psychological aspects, age, and BMI, on eating disorder psychopathology among adults with T1DM, with a particular focus on gender differences					
Wisting* (2018)	Norway	To assess the prevalence of DEB and associated symptoms of depression and anxiety among adult males and females with T1DM, investigate differences between individuals scoring below and above the cut-off on psychopathology, with a specific focus on metabolic control; and examine patterns of eating disorder psychopathology by age and weight					
Wisting* (2019; 75)	Norway	To investigate the psychometric properties and factor structure of the DEPS-R (Norwegian version) in adult males and females with T1DM					
Qualitative studies:							
McConnell (2001)	Ireland	To describe the difficulties frequently encountered with eating disorders and T1DM, with a description of four cases illustrating these points	4	1	Case Study Clinical Case Material	Local Diabetes Clinic Clinic-based sample	1 male reported misusing insulin in response to unpleasant situations
Morris (2021)	United Kingdom	Aim of the study is to explore the lived experience of diabulimia	6	1	Phenomenological Study Researcher-led semi-structured interviews with open-ended questions (Interpretative phenomenological analysis	Online Advertisement on an Academic Research Recruitment Platform, and the Social Media Platforms of two Diabetes Charities Population-based sample	1 male manipulated insulin for weight control -Commenced at the age of 25 years (2 years after his diagnosis) and continued for approximately 5 years
Roland (1982)	United Kingdom	To describe three patients with IDDM who developed anorexia nervosa	3	1	Case Study Clinical Case Material	The Royal Devon and Exeter Hospital and Exe Vale Hospital Clinic-based sample	1 male reported to have misused insulin for weight and shape
Sien (2020)	Malaysia	To determine factors of eating problems among a T1DM population	15	4	Phenomenological Study Researcher-led semi-structured interviews with open-ended questions (Thematic Analysis)	Outpatients of the University Malaya Medical Centre Clinic-based sample	No males were identified restricting and/or omitting insulin for the purpose of weight and shape control
Szmukler (1984)	United Kingdom	To describe the cases of six patients with IDDM and an eating disorder	6	1	Case Study Clinical Case Material	The Institute of Psychiatry Clinic-based sample	1 male reported to have misused insulin for weight and shape
Szmukler (1983)	United Kingdom	To report three cases of diabetes mellitus and anorexia nervosa and/or bulimia	3	1	Case Study Clinical Case Material	The Institute of Psychiatry Clinic-based sample	1 male reported to have misused insulin for weight and shape
Mixed methods:							
Boggiss (2020)	New Zealand/ United Kingdom	To examine the feasibility and acceptability of a brief self-compassion intervention for adolescents with T1DM and DEB	27	11	Randomised Feasibility Study DEPS-R self-report questionnaire and measures of feasibility and acceptability including open-ended questions (Content Analysis)	3 Paediatric and Adolescent Diabetes Clinics in New Zealand Clinic-based sample	At screening stage (n = 32 individuals with T1DM), across the past 28 days -15 males endorsed at least one item about omitting or restriction insulin a 1 or higher
Falcao (2017)	Portugal	To characterize and compare young adults with diabetes and their peers without diabetes on BMI, body image distortion and DEB, and to evaluate possible predictor variables of DEB in both groups; and to explore the perceptions of young adults with T1DM about its consequences for food, body image and weight, and to identify the use of insulin omission behaviour as a weight control strategy	55	18	Case-controlled Custom qualitative self-report questionnaire regarding deliberate insulin omission (Content Analysis)	Online advertisements published on social networks and groups and associations for people with diabetes Population-based sample	3 males omitted insulin intentionally, however, none of these males specifically reported that they did this intentionally to lose weight

^*^denotes the included studies that share the same dataset with at least one of the other included studies

*T1DM* type 1 diabetes mellitus, *T2DM* type 2 diabetes mellitus, *IDDM* insulin-dependent diabetes mellitus, *NIDDM* non-insulin-dependent diabetes mellitus, *DEB* disordered eating behaviour or disturbed eating behaviour, *AHEAD* Action for Health in Diabetes, *DEPS* Diabetes Eating Problem Survey, *DEPS-R* Diabetes Eating Problem Survey – Revised, *CBT* Cognitive Behavioural Therapy, *YEDE-Q* Youth Eating Disorder Examination Questionnaire, *EDI-3RC* Eating Disorder Inventory 3 Risk Composite, *chEDE* Child Eating Disorder Examination, *BMI* Body Mass Index, *zBMI* Body Mass Index (adjusted for age and sex), *HbA1c* glycated haemoglobin, *EAT-12* Eating Attitudes Test-12 item version, *EDE* Eating Disorders Examination, *EDI* Eating Disorder Inventory, *EDI-C* Eating Disorder Inventory for Children, *DSM-V* Diagnostic and Statistical Manual of Mental Disorders, 5th Edition, *EAT* Eating Attitudes Test, *EAT-36* Eating Attitudes Test – 36 item version, *BITE* Bulimic Investigatory Test, Edinburgh, *DSM-IV* Diagnostic and Statistical Manual of Mental Disorders, 4^th^ Edition, *FSE* Questionnaire for the Diagnosis of Eating Disorders, *SIAB* Structured Interview for Anorexic and Bulimic Nervosa, *MY-Q* MIND-Youth Questionnaire, *SCOFF* SCOFF Questionnaire

### Design

Most studies (*n* = 41) employed cross-sectional designs. Other designs included case studies (*n* = 4 studies), case–control (*n* = 4), longitudinal cohort (*n* = 3) and phenomenological designs (*n* = 2). One study employed a retrospective chart design [30], and another was a randomised feasibility trial [31]. Quantitative studies most commonly used a mixture of self-report questionnaires, variations of semi-structured, structured or diagnostic interviews and/or information about participants’ physical health (e.g., glycosylated haemoglobin [HbA1c], BMI etc.) collected from medical records or within clinic appointments (*n* = 46). Qualitative studies were predominantly case studies that described clinical case material [31–34]. The two mixed methods studies used self-report questionnaires [8, 31].

### Research focus

Most studies (n = 19) explored the prevalence of and/or correlates of disordered eating behaviours (including insulin restriction and/or omission) in individuals with T1DM [21, 35–52]. Three studies focused on sex differences in disordered eating behaviours in individuals with T1DM [22, 53, 54]. Others compared the nature of disordered eating among individuals with T1DM to individuals with T2DM (*n* = 4; 55–58) or "healthy" peers without diabetes mellitus (*n* = 8; 8, 23, 59–64), with one solely focusing on males [23]. Five studies focused on insulin restriction and/or omission for weight and/or shape control.

### Participant characteristics

All studies except for one [23] recruited male and female participants. Where possible, characteristics of the male participants were extracted separately or requested from authors (See Table 2).The total sample size of participants with T1DM across 45 studies was *n* = 12,450. Sample sizes varied widely from one (case studies) to 2,837 (*M* = 276.67, *SD* = 471.67). Across 43 studies, the total sample size of males with T1DM was 5,351 (*M* = 124.44, *SD* = 236). The mean age of males with T1DM across 21 studies was 19.67 years (*SD* = 2.87; *n* = 14) (see Supplementary Table 3 for the characteristics of all participants).

**Table 2:** Characteristics of Males with T1DM in Included Studies †

Variable	*N*_studies*_	*N*_*participants*_	*M* (SD)	Range
Males with T1DM				
*N*	43	5,351	124.44 (236)	1–1422
%	2	$\approx$420	51.3% (3.96)	48.5–54.1
Age of Males with T1DM				
M	21	2,873	19.67	
SD	14	2,817	(2.87)	
Range	2	470		9–19
Age Group	1	30		
Ethnicity (%)				
Males with T1DM	5	1,779	100%	
European/Caucasian	4	1, 257	70.66	
Australian	1	166	9.33	
Hispanic/Latino	1	171	9.61	
Black/African American	1	90	5.06	
Asian	1	20	1.12	
Native American	1	10	0.56	
Unknown	3	65	3.66	
Sex not separated	12	3,803		
Not supplied	28	4,984		

*N*_studies_ number of studies; *N*_participants_ number of participants; *M* Mean; *SD* standard deviation; *T1DM* type 1 diabetes mellitus;

^†^ not all studies provided this data for their participants

^*^based on *Nstudies* = 42 to avoid repetition of data presented across multiple studies

### Measures

Various measures were used to assess insulin restriction and/or omission in the context of weight and/or shape control, most of which were not psychometrically validated (See Table 1). Across the included studies, 24 studies used a measure that assessed insulin restriction and/or omission for weight and/or shape control [8, 21, 27, 35, 39, 40, 42, 43, 47–49, 51, 55–66]. The remaining studies asked about insulin restriction and/or omission broadly without identifying underlying reasons (*n* = 19; 22, 31, 36–38, 45, 50, 52–54, 67–75) or did not provide sufficient information to determine if reasons were accurately identified (*n* = 4; 23, 76–78). The measurement of insulin restriction and/or omission was unclear in nine studies [14, 32–34, 41, 44, 46, 79, 80]. Most commonly, studies measured engagement in the behaviour across the previous 28 days (*n* = 26 studies). The rating period across all included studies, however, varied from the previous seven days to ever, and in many studies (*n* = 16) the rating period was unclear.

### Quantitative, qualitative and mixed-methods findings

Across the included studies, the results for males with T1DM restricting and/or omitting insulin for weight and/or shape control were often combined with those of females. Where possible, data separated by sex were extracted and are presented below.

#### Quantitative Studies

##### Rates of insulin restriction and/or omission in males with T1DM.

Of the 33 quantitative papers, (25 samples), where insulin restriction and/or omission was identified among males with T1DM [21–23, 27, 35–39, 42, 43, 45, 50, 52–56, 58, 61, 65–70, 77], interpretable point estimate rates ranged from 1.4% [21, 35] to 76% [52–54, 75]. Fourteen quantitative studies reported no males restricting and/or omitting insulin for weight and/or shape control [30–32, 40, 41, 44, 46–49, 51, 59, 60, 64, 78]. One study reported only the percentage of males engaging in insulin restriction and/or omission who scored 20 or more on the Diabetes Eating Problems Survey – Revised (DEPS-R [73]; e.g., 37.5% and 25%, respectively [71]). One study reported suspicion of insulin restriction and/or omission in 2.4% of males, as determined by an expert panel [81].

##### Sex differences in insulin restriction and/or omission.

Many of the quantitative studies (*n* = 38*;* 21–22, 27, 30–32, 35–36, 39, 42–46, 48–51, 55–64, 67–71, 73–76, 81) reported insulin restriction and/or omission among female participants, with interpretable point estimates ranging between 5% [42] and approximately 75% [30]. Of these, differences between males and females were assessed in 12 samples. In Powers et al.’s study [45], females tended to report skipping insulin doses significantly more frequently than males. Females were also found to misuse insulin for weight and/or shape control more than males in Matthews [27] dissertation and in the study by Peducci et al. [43], however these differences were only marginally significant. In contrast, rates of insulin reduction or insulin omission between males and females with T1DM were not significant in studies by Troncone [68, 69]. The frequency of insulin omission and intentional reduction of insulin was also not significant [69]. Baechle et al. [67] similarly reported no significant difference between males and females when measuring insulin restriction at a frequency of at least three times per week and more than five times per week. Neither insulin restriction (or insulin restriction for weight and/or shape control was associated with sex in Beam et al. [66]. d'Emden et al. [39] also reported no difference when examining the presence of insulin manipulation or omission in males and females. Markowitz et al. [73] reported no difference between males and females regarding clinician-suspected insulin restriction or omission rates. While Bächle [sic] et al. [37] noted there were significantly more females than males who reported disordered eating behaviours using the SCOFF eating disorders screening questionnaire [82], insulin restriction did not significantly differ between males and females. In Baechle et al. [70], disordered eating symptom frequencies were consistently higher among females than males, except for insulin restriction. In Altinok et al. [71], while a greater proportion of females scored above the DEPS-R [73] cut-off, more males than females reported insulin restriction and insulin omission in this group. Finally, in the studies by Wisting et al. [52–54, 75], using the DEPS-R [73], males reported insulin restriction and insulin omission significantly more than females.

##### Frequency of insulin restriction and/or omission in males with T1DM.

Araia et al. [22] reported that across 14 days, approximately 14.4% of males misused insulin 1–3 days, and 3.8% of males misused insulin four or more days. Similarly, Baechle et al. [67] reported that 18.5% of males restricted insulin at least three times per week, with 6% restricting more than five times per week. In Beam et al.’s [66] study, males who endorsed insulin restriction for weight control reported this occurring 'rarely'. Insulin restriction occurring more than five times a week was used as a proxy for intentional insulin restriction as a disordered eating behaviour by Bächle [sic] et al. [37] and Baechle et al. [70], who reported approximately 7.25% and 8.2%, respectively, of males in their samples, deliberately restricted insulin respectively. Furthermore, Bächle [sic] et al. [37] reported that the mean number of carbohydrate units without insulin coverage per week for those who were SCOFF negative was 13.2 (*SD* = 10.6) and 18.8 (*SD* = 18.6) for those who were SCOFF positive. Troncone et al. [69], who used restriction or omitting at least one insulin shot per week as a proxy for intentional insulin restriction and/or omission, reported that 31.5% restricted insulin and 11.5% of males omitted insulin. Furthermore, Pursey et al. [61] reported that the mean frequency of insulin restriction in males across the past-28 days was 2.90 (*SD* = 7.245) compared to 1.67 (*SD* = 3.890) in females; the difference was not statistically significant. Males also had a larger range of days insulin was restricted across the past 28 days (0–28 days) than females (0–14 days) [61], indicating restriction and/or omission of insulin for weight control daily in one or more of the males. Similarly, while the mean score of insulin restriction using the DEPS-R ([73]; Item 4) ranged between "Rarely" and "Sometimes" for males [36, 52–54, 72, 75], answers ranged between 1 "Never" to 5 "Always" indicating some males are potentially engaging in the behaviour frequently. This was similar for insulin omission [36, 52–54, 72, 75], and ranged from 0 "Never" to 4 "Usually".

##### Measures of insulin restriction and/or omission among males with T1DM.

Insulin restriction and/or omission in males was identified more often when self-report measures were used compared to clinician-administered interviews. Phillipi et al. [44] was the only study using a self-report questionnaire that did not identify males misusing insulin, although exactly how insulin misuse was measured was unclear. Of the studies that used a clinician-administered interview, only two studies identified males misusing insulin (total of three males; 43, 55, 56). The remaining studies found no male participants misusing insulin [30–32, 47–49, 51, 59, 60, 64]. Similarly, researchers who used a clinician-administered interview and a self-report questionnaire either found no males reported insulin manipulation [40, 41, 46, 57] or found discrepancies in males' reports [23]. Interestingly, different rates of insulin restriction and/or omission were reported across two studies that shared the same sample of males [22, 36].

##### Correlates of insulin restriction and/or omission among males with T1DM.

Most included studies’ analyses combined male and female data. Of the included quantitative studies, seven reported correlates of insulin misuse. Wisting et al. [50] found that males who restricted insulin had significantly higher HbA1c than males not restricting insulin. However, Wisting et al. [51] found no significant associations between the pattern of eating and age, BMI, self-induced vomiting, binge eating, excessive exercise, intentional insulin omission, or metabolic control among males. Bächle [sic] et al. [37] in contrast reported that HbA1c values were highest across males who restricted insulin compared to males who did not report insulin restriction. Individuals restricting insulin had significantly worse average HbA1c values compared with individuals who were not restricting insulin, with HbA1c differences being greater among adolescent males than adolescent females [57]. One of the males misusing insulin in Svennson et al.’s [23] study had a high score on the Drive for Thinness subscale of the EDI-C [83]. Additionally, Araia et al. [22] found that body dissatisfaction was significantly correlated with eating binges and BMI for both sexes but was only significantly correlated with insulin omission for females. Among males in the study by Beam et al. [66], skipping insulin to lose weight was significantly correlated with HbA1c levels, diabetes self-management, overall difficulty with emotional regulation, difficulties with impulse control, limited access to strategies to regulate negative emotions, lack of clarity about what emotions are being experienced and symptoms of depression.

#### Qualitative studies

Across the six included qualitative studies, restriction and/or omission of insulin for weight and/or shape control was identified in four studies, with a total of four males [34–37]. Within these studies, males indicated a strong desire to lose weight [34, 62, 79, 80], and as described by one individual, insulin restriction and/or omission was akin to a "*magic pill*" ([62], p.62);

He expressed making a conscious decision to make his diabetes "*even more of a mess*" in pursuit of weight loss ([62], p. 74) and worth the sacrifice of poor health. Indeed, alongside the easy weight loss came a large physical and mental burden in the form of high blood glucose levels with blood sugars over 25 mmol/L [34], little energy [62], retinopathy [34, 62, 80], and feelings of regret, as one male described being "*half of everything [he] could have and should have been*" ([62], p. 67). One male engaging in insulin restriction reported welcoming feelings of "*queasiness*" experienced when his blood sugar levels were high, which diminished his appetite [79]. Another also described feeling better when he was a "*bit sugary*" [80]. He further expressed that he needed higher blood levels than others to feel well, which was also related to a dread of experiencing hypoglycaemia [80].

Interestingly, Morris [62] noted that the feelings of safety, belonging, and desirability that females gained through insulin restriction was not shared by the male participant. For this male, the behaviour was seen as a way of achieving better fitness performance through weight loss [62] and was used intermittently: *"It was not an ongoing thing; it was like a switch that I would turn off and turn back on"* ([62], p 74). In contrast to the male described by Morris [62], the males described by Szmukler and Russell [80] and Szmukler [79] reported the deliberate restriction and/or omission of insulin among other disordered eating behaviours (e.g., restriction of food intake, purging). McConnell et al.’s [33] case study reported the deliberate omission of insulin by a 9-year-old male in response to "*unpleasant situations*" rather than for weight and/or shape control, while Sien et al. [63] had no males who restricted and/or omitted insulin for weight and/or shape control.

#### Mixed methods studies

Falcao and Francisco [8] found that 11 participants with T1DM (20.8%) reported intentionally omitting insulin; of these, only three were males, and no males reported doing it intentionally to lose weight. Therefore, the qualitative data concerning insulin restriction and/or omission for weight and/or shape control did not contain any males with T1DM. Of the 32 adolescents screened for inclusion by Boggiss et al. [31], 15 males (46.9% of the total sample) responded to at least one item about omitting or restricting insulin (either item 4 ‘when I overeat, I don’t take enough insulin to cover the food’ or item 13 ‘after I overeat I skip my next insulin dose) with a 1 (rarely) or higher versus 13 females.

## Discussion

### Main findings and implications

Our scoping review, the first of its kind, suggests that the deliberate restriction and/or omission of insulin is a unique disordered eating behaviour that may occur at comparable rates in males with T1DM to that of females with T1DM. Where it was reported to occur, estimates of the behaviour ranged from 5% [42] to 75% [30] in females and 1.4% [21, 35] to 76% in males [52–54, 75]. Across 12 samples [27, 37, 39, 43, 45, 52, 54, 66–71, 73, 75] where sex differences were examined, higher prevalence in females was reported in only three [27, 43, 45], suggesting sex differences may not be as great as earlier thought. Furthermore, our synthesis summarises what is currently known about the behaviour among males with T1DM, including correlates, and provides insights into limitations and directions for future research, as well as clinical recommendations. Our review also identifies several areas of concern about how researchers conduct and report results on insulin restriction and/or omission in males with T1DM.

Consistently, there is a marginalisation of males with T1DM within research exploring this dangerous behaviour and a paucity of research examining the nature and relationships of insulin restriction and/or omission for weight and/or shape control in males with T1DM. It is plausible that research in this area may be somewhat stunted by the idea that disordered eating behaviours occur much less often in males with T1DM and males generally than in females [84, 85]. Nonetheless, our review demonstrates sufficient evidence exists to suggest that this dangerous behaviour may be a significant problem for males and that it warrants attention in the literature especially given the severe consequences it poses to their morbidity and mortality [4, 15].

Of the included studies, many also did not provide data on the relationships of insulin restriction and/or omission with other variables among males. This omission made it difficult to understand why males may or may not deliberately restrict and/or omit insulin for weight and/or shape control and how it impacts males. While current evidence is limited, the driving motivators for insulin restriction and/or omission for weight and/or shape control may be qualitatively different for males than females. Findings suggest that reasons beyond body dissatisfaction may drive the behaviour among males with T1DM, including a desire to improve fitness performance [11] or in response to negative affect and difficulty regulating emotion [66]. Both negative affect generally and negative diabetes-related affect have previously been associated with increased odds of restricting insulin [86]. However, our results suggest differences exist in how emotion dysregulation relates to insulin restriction and/or omission for weight and/or shape control between males and females. Health professionals may wish to explore motivators for the behaviour as males and females with T1DM may be motivated by different factors. There have been previous calls for researchers to identify the motivation underlying insulin restriction more carefully [87], as individuals with T1DM may restrict and/or omit insulin due to fear of injections, fear of hypoglycaemia, denial of disease and avoidance of injections in social settings [32].

Diverse and generally poor assessment of insulin restriction and/or omission for weight and/or shape control also limited our review. Our findings identified the need for a standardised measure of insulin restriction and/or omission for weight and/or shape control while considering differences in males’ and females’ prescribed body ideals. Researchers’ use of customised or adapted questionnaires to measure insulin misuse is also highlighted and may be due to the limited data on effective screening tools. Standard questionnaires for disordered eating behaviours and eating disorders for individuals with T1DM have been criticised for under- and over-estimating disordered eating behaviours [3, 73]. While currently the most validated tool for adolescents and adults with T1DM, the DEPS-R [73, 88] does not directly enquire about insulin misuse for weight and/or shape control. The DEPS-R [73] and other existing tools for measuring body dissatisfaction, body image concerns and disordered eating behaviours are also often female-centric and less geared towards concerns more commonly held by males [84]. Furthermore, there are often high levels of secrecy and shame around disordered eating behaviours and eating disorders including insulin misuse [11, 27, 89–91], and individuals may be secretive about their engagement in disordered eating behaviours for fear of stigmatisation [92, 93] or due to low motivation for change [94]. Against this backdrop, identifying insulin restriction and/or omission generally among people with T1DM is difficult; however, our results suggest that a self-report measure capturing insulin restriction and/or omission may facilitate more honest reporting by individuals and an increased willingness to disclose the behaviour. In the absence of a psychometrically robust measure, we suggest that health professionals use a validated, self-report measure to explore the presence of body dissatisfaction and disordered eating behaviours, followed by open-ended questions to explore current and past deliberate restriction and/or omission of insulin among their patients. Table 3 outlines possible measures for use among males with T1DM, alongside sensitive open-ended questions to gather information about insulin use and weight and shape concerns.

**Table 3:** Suggested validated, self-report measures and sensitive, open-ended questions for use among Males with T1DM

Self-Report Measures •The Body Mass Index Silhouette Matching Test (BMI-SMT; 98) •The Male Body Attitudes Scale (MBAS; 99) •The male version of the Sociocultural Attitudes Toward Appearance Questionnaire – 4 (SATAQ-4R-Male; [95])
Weight and Shape Questions •How do you feel about your weight and/or shape? •How satisfied are you with your weight and/or shape? •How does your weight and/or shape affect the way you feel about yourself? •How important is it to you to change your weight and/or shape? •How would you want to change your weight and/or shape? •How would you go about changing your weight and/or shape? •What behaviours might you engage in to change your weight and/or shape?
Insulin Use Questions •Some people with diabetes find it difficult to manage their insulin treatment. How is this going for you? •Your blood sugar levels have been elevated recently. What do you think may be the reason/s for this increase? •Could you tell me about the reasons you skip or reduce insulin?

Our review was limited to studies in English, and we excluded studies examining the behaviour in T2DM due to different aetiology and management aspects compared to T1DM. Also, possibly eligible studies were excluded where male data were not reported separately, and we received no response from the authors when we sought these data. The included studies were heterogeneous and could not be directly compared statistically, which limited interpretation. In addition, as included studies primarily used cross-sectional designs, we could not make inferences about cause and effect. Most included studies focused on individuals aged in early adulthood, which hinders the generalisability of the results to other age groups. The included studies did not provide information on males' sexual or gender identity, and therefore little can be concluded on how this may be related to insulin misuse. Additionally, future research comparing males with and without diabetes and employing longitudinal study designs is needed to explore contributing factors and trajectories of disordered eating in diabetes.

Most studies tended not to include comparison groups of males without T1DM, so it is unclear to what extent the explored factors are relevant to males with T1DM only or also to other male populations. Many participants were recruited from a clinic or specialist centre and therefore may not be representative of the larger population of individuals with T1DM. Also, we cannot rule out possible selection bias. It may be that non-respondents are more likely to engage in insulin restriction and/or omission for weight and/or shape control or have more severe symptoms.

Given that data for males and females with T1DM was most commonly combined, we recommend future research report data separately by sex [96]. Also, more precise terminology should be used when reporting insulin restriction and/or omission among males and females with T1DM, as different definitions reflect different behaviours [4, 88, 97]. Additionally, as males are poorly represented in qualitative research, with reports from only four men included in our review, we recommend further qualitative research to gain males’ experiential perspectives of insulin restriction and/or omission for weight and/or shape to better understand behavioural aetiology and correlates.

## Conclusions

Previously thought to occur much more frequently in females with T1DM, our results suggest that males with T1DM engage in insulin restriction and/or omission for weight and/or shape control at similar rates. Health professionals should be alert to the potential for its presence in both sexes and should employ a self-report measure to explore the presence of body dissatisfaction and disordered eating behaviours, followed by open-ended questions to inquire about the presence of insulin restriction and/or omission for weight and/or shape control. This vigilance is particularly critical given the increased risk of morbidity and mortality this behaviour poses to individuals, regardless of sex.

## Supplementary Information

Below is the link to the electronic supplementary material.

Supplementary file1 (DOCX 35 KB)

## Data Availability

All data is contained within the manuscript file and its additional files.

## Declarations

### Public significance statement

This review is the first to summarise existing literature on the deliberate restriction and/or omission of insulin as a disordered eating behaviour in males with type 1 diabetes mellitus. The results highlight several concerns in the research thus far, demonstrate the need for further research on the behaviours' aetiology and impact within males and highlights the need for health professionals to be aware of its presence in males and females.
