# Supplementary material for: The misuse of insulin by males with Type 1 Diabetes Mellitus for weight and/or shape control: a systematic scoping review
Source: J Diabetes Metab Disord. 2022 Nov 15;22(1):13–34. doi: 10.1007/s40200-022-01151-8 (PMC10225459; doi:10.1007/s40200-022-01151-8)
Supplement: Supplementary file 1 — Supplementary file1 (DOCX 35 KB) [file 40200_2022_1151_MOESM1_ESM.docx]

Supplementary Table 1: Preferred Reporting Items for Systematic reviews and Meta-Analyses extension for Scoping Reviews (PRISMA-ScR) Checklist

| **SECTION** | **ITEM** | **PRISMA-ScR CHECKLIST ITEM** | **REPORTED ON PAGE #** |
| --- | --- | --- | --- |
| **TITLE** | | | |
| Title | 1 | Identify the report as a scoping review. | 1 |
| **ABSTRACT** | | | |
| Structured summary | 2 | Provide a structured summary that includes (as applicable): background, objectives, eligibility criteria, sources of evidence, charting methods, results, and conclusions that relate to the review questions and objectives. | 3 |
| **INTRODUCTION** | | | |
| Rationale | 3 | Describe the rationale for the review in the context of what is already known. Explain why the review questions/objectives lend themselves to a scoping review approach. | 4-6 |
| Objectives | 4 | Provide an explicit statement of the questions and objectives being addressed with reference to their key elements (e.g., population or participants, concepts, and context) or other relevant key elements used to conceptualize the review questions and/or objectives. | 5-6 |
| **METHODS** | | | |
| Protocol and registration | 5 | Indicate whether a review protocol exists; state if and where it can be accessed (e.g., a Web address); and if available, provide registration information, including the registration number. | 5-6 |
| Eligibility criteria | 6 | Specify characteristics of the sources of evidence used as eligibility criteria (e.g., years considered, language, and publication status), and provide a rationale. | 6-7 |
| Information sources* | 7 | Describe all information sources in the search (e.g., databases with dates of coverage and contact with authors to identify additional sources), as well as the date the most recent search was executed. | 6-7 |
| Search | 8 | Present the full electronic search strategy for at least 1 database, including any limits used, such that it could be repeated. | Supplementary Table 2 |
| Selection of sources of evidence† | 9 | State the process for selecting sources of evidence (i.e., screening and eligibility) included in the scoping review. | 6-7 |
| Data charting process‡ | 10 | Describe the methods of charting data from the included sources of evidence (e.g., calibrated forms or forms that have been tested by the team before their use, and whether data charting was done independently or in duplicate) and any processes for obtaining and confirming data from investigators. | 7 |
| Data items | 11 | List and define all variables for which data were sought and any assumptions and simplifications made. | 7 |
| Critical appraisal of individual sources of evidence§ | 12 | If done, provide a rationale for conducting a critical appraisal of included sources of evidence; describe the methods used and how this information was used in any data synthesis (if appropriate). | N/A |
| Synthesis of results | 13 | Describe the methods of handling and summarizing the data that were charted. | 7 |
| **RESULTS** | | | |
| Selection of sources of evidence | 14 | Give numbers of sources of evidence screened, assessed for eligibility, and included in the review, with reasons for exclusions at each stage, ideally using a flow diagram. | 7 & Figure 1 |
| Characteristics of sources of evidence | 15 | For each source of evidence, present characteristics for which data were charted and provide the citations. | 7-12, Table 1 & Reference List |
| Critical appraisal within sources of evidence | 16 | If done, present data on critical appraisal of included sources of evidence (see item 12). | N/A |
| Results of individual sources of evidence | 17 | For each included source of evidence, present the relevant data that were charted that relate to the review questions and objectives. | 7-12 & Table 1 |
| Synthesis of results | 18 | Summarize and/or present the charting results as they relate to the review questions and objectives. | 7-12 |
| **DISCUSSION** | | | |
| Summary of evidence | 19 | Summarize the main results (including an overview of concepts, themes, and types of evidence available), link to the review questions and objectives, and consider the relevance to key groups. | 12-15 |
| Limitations | 20 | Discuss the limitations of the scoping review process. | 14-15 |
| Conclusions | 21 | Provide a general interpretation of the results with respect to the review questions and objectives, as well as potential implications and/or next steps. | 15 |
| **FUNDING** | | | |
| Funding | 22 | Describe sources of funding for the included sources of evidence, as well as sources of funding for the scoping review. Describe the role of the funders of the scoping review. | 16 |

PRISMA-ScR = Preferred Reporting Items for Systematic reviews and Meta-Analyses extension for Scoping Reviews.

Supplementary Table 2: *Search Strategy*

| Insulin Misuse | Weight/Shape Control | Males | T1DM |
| --- | --- | --- | --- |
| PubMED |  |  |  |
| "insulin"[mh] OR insulin*[tiab] OR insulin resistance[tiab] OR insulin purging[tiab] OR insulin misuse[tiab] OR insulin omission[tiab] OR insulin restriction[tiab] | "diabulimia"[mh] OR "binge-eating disorder"[mh] OR "anorexia nervosa"[mh] OR "body dysmorphic disorders"[mh] OR "anorexia"[mh] OR "feeding and eating disorders"[mh:noexp] OR "feeding and eating disorders of childhood"[mh] OR "bulimia nervosa" [mh] OR "bulimia"[mh] OR "body image"[mh] OR purging disorder*[tiab] OR binge-eating*[tiab] OR bingeing[tiab] OR anorexia*[tiab] OR diabulimia [tiab] OR bulimia*[tiab] OR eating disorder*[tiab] OR disordered eating*[tiab] OR eating disorder not otherwise specified[tiab] OR  EDNOS[tiab] OR body dysmorphic disorder*[tiab] OR body image[tiab] OR body image disorder*[tiab] OR weight control[tiab] OR weight management[tiab] OR body ideal[tiab] OR body shape[tiab] OR shape control[tiab] | "male"[mh] OR "men"[mh] OR male*[tiab] OR boy[tiab] OR boys[tiab] OR men[tiab] OR mens[tiab] OR human male[tiab] | "diabetes mellitus, type 1" [mh] OR "diabetes mellitus"[mh] OR diabetes mellitus type 1[tiab] OR type 1 diabetes[tiab] OR type 1 diabetic*[tiab] OR insulin dependent diabetes mellitus[tiab] OR diabetes mellitus, insulin-dependent[tiab] OR insulin-dependent diabetes mellitus[tiab] OR diabetes mellitus, juvenile-onset[tiab] OR juvenile-onset diabetes mellitus[tiab] OR IDDM[tiab] OR type 1 diabetes[tiab] OR diabetes, type 1[tiab] OR diabetes, autoimmune[tiab] |
| PsycINFO |  |  |  |
| Insulin.sh OR (insulin adj4 (omit* OR omission* OR resistance OR purge OR purging OR misuse* OR restrict*)).tw | Body image.sh OR body esteem.sh OR body image disturbances.sh OR body dysmorphic disorder.sh OR exp eating disorders OR weight control.sh OR purging disorder*.tw. OR binge-eating*.tw. OR bing*.tw. OR anorexi*.tw OR diabulimi*.tw. OR bulimi*.tw. OR eating disorder*.tw. OR disordered eating*.tw OR "eating disorder not otherwise specified".tw OR EDNOS.tw OR body image disturbance*.tw. OR body dysmorphic disorder*.tw. OR body image.tw. OR weight control.tw. OR weight management.tw. OR body ideal.tw. OR body shape.tw. OR shape control.tw. | Human males.sh OR male*.tw. OR men.tw. OR mens.tw OR boy.tw. OR boys.tw. | Exp Diabetes OR diabetes mellitus type 1.tw. OR (diabet* adj4 type 1).tw. OR insulin dependent diabetes mellitus.tw. OR diabetes mellitus, insulin-dependent.tw. OR diabetes mellitus, juvenile-onset.tw. OR juvenile-onset diabetes mellitus.tw. OR IDDM.tw. OR diabetes, autoimmune.tw. |
| Web of Science |  |  |  |
| "insulin" OR "insulin omission" OR "insulin resistance" OR "insulin purging" OR "insulin misuse" OR "insulin restriction" | "binge-eating disorder*" OR "anorexia*" OR "feeding and eating disorders" OR "feeding and eating disorders of childhood" OR "bulimia*" OR "eating disorder*" OR "purging disorder" OR "diabulimia" OR "bingeing" OR "disordered eating*" OR "eating disorder not otherwise specified" OR "EDNOS" OR "body dysmorphic disorder*" OR "body image" OR "body image disorder*" OR "weight control" OR "weight management" OR "body ideal" OR "body shape" OR "shape control" | "male*" OR "men" OR "mens" OR "boy" OR "boys" OR "human male*" | "diabetes mellitus type 1" OR "diabetes" OR "type 1 diabetes" OR "type 1 diabetic*" OR "insulin dependent diabetes mellitus"  OR "diabetes mellitus, insulin-dependent" OR "insulin-dependent diabetes mellitus" OR "diabetes mellitus, juvenile-onset" OR "juvenile-onset diabetes mellitus" OR "IDDM" OR "type 1 diabetes" OR "diabetes, type 1" OR "diabetes, autoimmune" |
| CINAHL |  |  |  |
| MH insulin+ OR (insulin N4 (omit* OR omission* OR resistance OR purge OR purging OR misuse* OR restrict*)) | MH "binge-eating disorder+" OR  MH "anorexia nervosa+" OR MH anorexia+ OR MH "feeding and eating disorders+" OR MH "feeding and eating disorders of childhood+" OR MH "bulimia nervosa+" OR MH bulimia+ OR MH "eating disorders+" OR MH "body dissatisfaction+" OR MH "purging disorder+" OR MH "body image+" OR MH "weight control+" OR MH "weight loss+" OR TI "binge eating disorder*" OR AB "binge eating disorder*" OR TI anorexi* OR AB anorexi* OR TI "bulimia nervosa" OR AB "bulimia nervosa" OR TI "eating disorder*" OR AB "eating disorder*" OR TI "appetite disorder*" OR AB "appetite disorder*" OR TI diabulimi* OR AB diabulimi* OR TI "disordered eating*" OR AB "disordered eating*" OR TI "purging disorder*" OR AB "purging disorder*" OR TI purging OR AB purging OR TI EDNOS OR AB EDNOS OR TI "eating disorder not otherwise specified" OR AB "eating disorder not otherwise specified" OR TI "body dysmorphic disorder*" OR AB "body dysmorphic disorder*" OR TI "body image" OR AB "body image" OR TI "body image disorder*" OR AB "body image disorder*" OR TI "weight control" OR AB "weight control" OR TI "weight management" OR AB "weight management" OR TI "body ideal" OR AB "body ideal" OR TI "body shape" OR AB "body shape" OR TI "shape control" OR AB "shape control" | MH male+ OR TI male* OR AB male* OR TI men OR AB men OR TI mens OR AB mens OR TI boy OR AB boy OR TI boys OR AB boys OR TI "human male*" OR AB "human male*" | MH "diabetes mellitus+" OR MH diabetes+ OR TI "diabetes mellitus, type 1" OR AB "diabetes mellitus type 1" OR TI "type 1 diabetes mellitus" OR AB "type 1 diabetes mellitus" OR TI "type 1 diabetes" OR AB "type 1 diabetes" OR TI "type 1 diabetic*" OR AB "type 1 diabetic*" OR TI "insulin dependent diabetes mellitus" OR AB "insulin dependent diabetes mellitus" OR TI "diabetes mellitus, insulin-dependent" OR AB "diabetes mellitus, insulin-dependent" OR TI "insulin-dependent diabetes mellitus" OR AB "insulin-dependent diabetes mellitus" OR TI "diabetes mellitus, juvenile-onset" OR AB "diabetes mellitus, juvenile-onset" OR TI "juvenile-onset diabetes mellitus" OR AB "juvenile-onset diabetes mellitus" OR TI IDDM OR AB IDDM OR TI "type 1 diabetes" OR AB "type 1 diabetes" OR TI "diabetes, type 1" OR AB "diabetes, type 1" OR TI "diabetes, autoimmune" OR AB "diabetes, autoimmune" |
| Embase |  |  |  |
| insulin/exp OR  "insulin omission":ti,ab OR "insulin resistance":ti,ab OR "insulin purging":ti,ab OR "insulin restriction":ti,ab OR "insulin misuse":ti,ab | "binge eating disorder"/exp OR "anorexia nervosa"/exp OR bulimia/exp OR "eating disorder"/exp OR "purging disorder"/exp OR "body weight control"/exp OR "purging disorder*":ti,ab OR binge-eating:ti,ab OR bingeing:ti,ab OR anorexia*:ti,ab OR diabulimia:ti,ab OR bulimia*:ti,ab OR "eating disorder*":ti,ab OR "disordered eating*":ti,ab OR "eating disorder not otherwise specified":ti,ab OR EDNOS:ti,ab OR "body dysmorphic disorder*":ti,ab OR "body image":ti,ab OR "body image disorder*":ti,ab OR "weight control":ti,ab OR "weight management":ti,ab OR "body ideal":ti,ab OR "body shape":ti,ab OR "shape control":ti,ab | male/exp OR men:ti,ab OR mens:ti,ab OR boy:ti,ab OR boys:ti,ab OR "human male*":ti,ab | "diabetes mellitus"/exp OR "diabetes"/exp OR "diabetes mellitus, type 1":ti,ab OR "type 1 diabetes":ti,ab OR "type 1 diabetic*":ti,ab OR "insulin dependent diabetes mellitus":ti,ab OR "diabetes mellitus, insulin-dependent":ti,ab OR "insulin-dependent diabetes mellitus":ti,ab OR "diabetes mellitus, juvenile-onset":ti,ab OR "juvenile-onset diabetes mellitus":ti,ab OR "IDDM":ti,ab OR "type 1 diabetes":ti,ab OR "diabetes, type 1":ti,ab OR "diabetes, autoimmune":ti,ab |
| Scopus |  |  |  |
| TITLE-ABS-KEY (insulin OR "insulin omission" OR "insulin resistance" OR "insulin purging" OR "insulin misuse" OR "insulin restriction") | TITLE-ABS-KEY ("binge-eating disorder*" OR anorexia* OR "feeding and eating disorders" OR "feeding and eating disorders of childhood" OR bulimia* OR "eating disorder*" OR "purging disorder" OR diabulimia OR bingeing OR "disordered eating*" OR "eating disorder not otherwise specified" OR EDNOS OR "body dysmorphic disorder*" OR "body image" OR "body image disorder*" OR "weight control" OR "weight management" OR "body ideal" OR "body shape" OR "shape control") | TITLE-ABS-KEY (male* OR men OR mens OR boy OR boys OR "human male*") | TITLE-ABS-KEY ("diabetes mellitus type 1" OR diabetes OR "type 1 diabetes" OR "type 1 diabetic*" OR "insulin dependent diabetes mellitus" OR "insulin dependent diabetes mellitus"  OR "diabetes mellitus, insulin-dependent" OR "insulin-dependent diabetes mellitus" OR "diabetes mellitus, juvenile-onset" OR "juvenile-onset diabetes mellitus" OR "IDDM" OR "type 1 diabetes" OR "diabetes, type 1" OR "diabetes, autoimmune") |

Supplementary Table 3: *Characteristics of Participants (Men and Women with T1DM) in Included Studies* †

| *Variable* | *N*_studies*_ | *N_participants_* | *M* (SD) | *Range* |
| --- | --- | --- | --- | --- |
| Participants with T1DM |  |  |  |  |
| *N* | 45 | 12,450 | 276.67 (471.67) | 1-2837 |
| Age |  |  |  |  |
| *M* | 37 | 11,961 | 19.21 |  |
| *SD* | 33 | 10,866 | (3.92) |  |
| *Range* | 28 | 7,050 |  | 2.8-79 |
| *Age Group* | 3 | 1,052 |  |  |
| Ethnicity (%) |  |  |  |  |
| Total Sample | 17 | 7,466 | 100% |  |
| *European/Caucasian* | 12 | 3,752 | 30.14 |  |
| *Australian* | 2 | 487 | 3.91 |  |
| *Hispanic/Latino* | 4 | 431 | 3.47 |  |
| *Black/African American* | 6 | 314 | 2.52 |  |
| *Asian* | 4 | 72 | 0.58 |  |
| *Brazilian* | 1 | 189 | 1.52 |  |
| *Native American* | 1 | 21 | 0.19 |  |
| *Polynesian* | 1 | 6 | 0.05 |  |
| *Unknown* | 13 | 2,194 | 17.62 |  |
| Not supplied | 28 | 4,984 |  |  |

*Note*. *N*_studies_ = number of studies; *N*_participants_ = number of participants; *M* = Mean; *SD* = standard deviation; T1DM = type 1 diabetes mellitus

† not all studies provided this data for their participants.

*based on *Nstudies* = 45 to avoid repetition of data presented across multiple studies
